# Cdk activation by phosphorylation: linking growth signals to cell cycle control

**DOI:** 10.1042/BST20253004

**Published:** 2025-05-09

**Authors:** Heidi M. Blank, Eun-Gyu No, Michael Polymenis

**Affiliations:** Department of Biochemistry and Biophysics, Texas A&M University, 300 Olsen Blvd., College Station, Texas 77843, U.S.A.

**Keywords:** CAK, Cak1p, Cdc28p, cell cycle, phosphorylation, T-loop

## Abstract

Cells adjust their proliferation in response to extrinsic factors and nutrients. Such inputs must reach the cell cycle machinery to ensure proper cell proliferation. This minireview focuses on evidence suggesting that phosphorylating the T-loop domain of cyclin-dependent kinases may be a critical and conserved conduit for these external signals. Understanding this regulatory mechanism could provide crucial insights into how all eukaryotic cells integrate external information to decide whether or not to divide.

## The activating phosphorylation of cyclin-dependent kinases

Cyclin-dependent kinases (Cdks) drive cell cycle transitions in all eukaryotes [1,2]. The active protein kinase enzymes are complexes of a catalytic (Cdk) and a regulatory (cyclin) subunit. Cyclins are necessary for Cdk activity because they change the Cdk’s conformation and allow it to bind its substrates [3]. Without cyclin, a part of the Cdk called the T-loop and a small helix next to it block the active site of the Cdk, and substrates cannot access it [4]. The T-loop is analogous to the regulatory or activation loop of other eukaryotic protein kinases [5]. Upon cyclin binding, the T-loop moves, and the obstructing helix changes to a strand, making the active site more accessible to substrates. It also changes the conformation of the ATP-binding site of the Cdk, allowing ATP to bind correctly, and aligns critical catalytic residues for efficient phosphate transfer.

However, more conformational changes are needed for Cdk activation. A conserved threonine in the T-loop (T169 in the budding yeast Cdc28p, T160 in human Cdk2) must be phosphorylated for additional conformational changes in the active site, allowing productive substrate binding. The negative charge of the phosphate group on the T-loop is neutralized by its interactions with three arginine residues through a network of hydrogen bonds [6]. This network of hydrogen bonds is extensive, affecting parts of the Cdk and the cyclin [6]. The phosphorylated T-loop moves by about 7 Å, further opening the substrate-binding site, allowing for additional cyclin–Cdk contacts, and further stabilizing the active conformation of the kinase [6]. In some cases, such as the human Cdk4–cyclinD1 complex, substrate binding brings about additional conformational changes necessary for full activation [7].

T-loop phosphorylation adds to the list of ways to control Cdk activity, including by binding to other proteins and phosphorylation at other residues of the Cdk [1]. The outcomes could be activation (e.g., cyclin binding, binding to non-canonical activators [8], T-loop phosphorylation), inhibition (e.g., N-terminal Tyr phosphorylation, binding to protein inhibitors), or other changes (e.g., enhanced processivity by binding to Cks1p, altered specificity via docking interactions between substrates and cyclins). The numerous layers of Cdk control reflect the Cdk’s central role in governing cell proliferation.

Although T-loop phosphorylation is essential for Cdk activity, a phospho-mimicking mutation (T169E in the budding yeast Cdc28p) is also lethal in yeast [9]. But if additional Cdk mutations are introduced, then the Cdk T169E mutant supports normal cell cycle progression in yeast [9]. The mutations were far from T169, suggesting that global structural changes rather than a localized perturbation of the phosphothreonine binding pocket were responsible for their suppressing ability [9]. These results also showed that, unlike other layers of Cdk control that are periodic in the cell cycle (e.g., cyclin binding and inhibitory N-terminal phosphorylation), at least in budding yeast, the Cdk does *not* need to be periodically phosphorylated and dephosphorylated at the T-loop. In human HAP1 cells (a near-haploid leukemia cell line), mutating the T161 of Cdk1 to the non-phosphorylatable T161A reduced cell proliferation rates [10]. On the other hand, cells carrying the phospho-mimicking T161E mutation proliferated normally, which was interpreted as a ‘lack of requirement of regulation of Cdk-activating kinase (CAK) in human cells’ [10]. How do cells then use T-loop phosphorylation to control Cdk activity?

## The different CAKs

The kinases responsible for the T-loop activating phosphorylation are called CAKs. CAK-dependent phosphorylation is conserved from yeast to humans [11]. All CAKs are members of the large Cdk family [12], which includes Cdks with non-cell cycle roles, such as those involved in transcriptional control. There are significant differences among CAKs of different species. Budding yeast Cak1p is the only CAK in yeast [11], and T169 phosphorylation is the only essential function of Cak1p [9]. The closest relative and true ortholog of Cak1p is the fission yeast Csk1 protein [12], which also functions as CAK [13]. However, fission yeast has two CAK activities. Csk1’s CAK role is shared by Mcs6 [13], another member of the large Cdk family [12]. Mcs6 belongs to the Cdk group that has roles in transcription and targeting RNA polymerase [12]. The budding yeast Mcs6 ortholog, Kin28p, targets RNA polymerase but has no CAK activity. In plants, the situation resembles that in fission yeast, having both types of CAK activity, one resembling budding yeast Cak1p [14] and another with transcriptional roles [15]. In metazoans, however, which do not have Cak1p or Csk1 orthologs [12], the Cdk7–cyclinH transcriptional Cdk complex provides the only CAK function known so far [16,17]. Active Cdk7–cyclinH dimers can form if Cdk7 is phosphorylated at its T-loop, but the MAT1 assembly factor can bypass this requirement [18]. Cdk7–cyclinH can also be part of a larger complex, the transcription factor IIH (TFIIH) [19], which includes several additional subunits, including the XPD and XPB helicases. TFIIH phosphorylates proteins with transcriptional roles, including the C-terminal domain (CTD) of RNA Polymerase II, a function necessary for transcription activation. Note that budding yeast Cak1p is not a component of TFIIH and does not target the CTD [20]. Finally, whereas mammalian Cdk inhibitors of the CIP/KIP (p21, p27, p57) and the INK4 (p16, p18) classes block the CAK activity of MAT1/Cdk7–cyclinH, Cdk inhibitors do not block yeast Cak1p [20].

There are also different substrate specificities among the different CAKs. Budding yeast Cak1p, fission yeast Csk1, and *Arabidopsis thaliana* CAKAT (also called CDKF) prefer to target the Cdk monomer [20,21]. In certain cases, however, yeast Cak1p can preferentially target non-yeast Cdk–cyclin dimers [22]. Human CAK targets some Cdks as monomers and others as Cdk–cyclin dimers. Human cells have several Cdks with cell cycle roles, and the Cdk7–cyclinH CAK phosphorylates and activates them. Early in the cell cycle, in the G1 phase, the Cdk7–cyclinH CAK phosphorylates Cdk4/6–cyclinD complexes, triggering the G1/S transition [23]. Cdk2 and Cdk1, which function later, are also targeted by Cdk7–cyclinH CAK. But while Cdk7–cyclinH CAK can phosphorylate the Cdk2 monomer, it only phosphorylates Cdk1 when Cdk1 is bound to a cyclin [24]. These differences toward Cdk2 and Cdk1 help to establish the Cdk–cyclin pairing order. In S phase, Cdk2 pairs with cyclinA. But Cdk1 also binds cyclinA. Since the Cdk7–cyclinH CAK targets the Cdk2 monomer but only the Cdk1/−cyclin dimer, it helps ensure that Cdk2–cyclinA complexes will be activated before Cdk1–cyclinA ones [24].

## Regulation of CAK and T-loop phosphorylation

### Budding yeast Cak1p

Perhaps surprisingly for a regulator critical for the activity of the cell division machinery, the abundance, activity, or localization of Cak1p are *not* regulated in continuously dividing cells in rich media [11,25]. There is also no evidence that Cak1p is modified post-translationally, and mutating putative phosphorylation sites on Cak1p does not affect its activity [25]. Cak1p is a very stable protein, with no observable degradation after 3 hours in cells dividing vegetatively [25] and an estimated half-life of more than 16 hours [26]. Since, in standard laboratory conditions, the duration of each cell cycle is much shorter than the half-life of Cak1p, it is not surprising that there is no evidence for periodic changes in the abundance of Cak1p. Thus far, the only evidence for some type of control of Cak1p levels in vegetative cells is in the stationary phase, where the abundance of Cak1p drops significantly [25].

Although our focus here is on the role of CAK in Cdk T-loop phosphorylation in mitotically dividing cells, the levels of Cak1p are dynamic in meiosis [25,26]. Cak1p is required early in the meiotic program for DNA synthesis [27]. After gamete generation, Cak1 is also required for spore wall morphogenesis [28]. It directly phosphorylates the Smk1p kinase at its activation loop [29], activating Smk1p and the developmental program needed for spore formation.

### Metazoan CAK and T-loop phosphorylation changes

The levels and activity of Cdk7 in the CAK complex do not change in the cell cycle of synchronous, exponentially dividing human (HeLa) cells [30], normal lymphocytes [31], or in frog oocytes undergoing early embryonic cleavages [32]. An interesting case (exception?) of CAK regulation has been shown in flies, where an excess of the XPD DNA helicase TFIIH subunit moderately lowers CAK activity but significantly reduces viability and impairs mitotic progression [33]. XPD titrates Cdk7–cyclinH away from the nucleus and its CAK roles [33]. Conversely, at the beginning of mitosis, XPD is degraded, and CAK can now move into the nucleus and activate Cdk1, promoting mitosis [33]. Other proteins, such as MMS19, can also bind and sequester XPD away from CAK, freeing CAK complexes to phosphorylate and activate Cdk1 [34]. It remains unclear whether the above XPD-dependent mode of CAK control operates in other systems. Instead, it is thought that CAK activity is not regulated in most other contexts of continuously dividing cells in constant environments [17].

Furthermore, imposing various cell cycle blocks does not change CAK activity in metazoan systems [35]. Cdk7 levels, however, are higher in many tumor-derived human cell lines [31], and the selective Cdk7 inhibitor samuraciclib is currently evaluated in several clinical trials (NCT05963997, NCT05963984, NCT06125522). There are conflicting reports about the levels of Cdk7 in quiescent cells, where, in some cases, there is a drop [32] but not in others [31]. Overall, the lack of significant periodic changes in the levels and activity of metazoan CAK is similar to the situation in budding yeast [11,25,36], leading to the puzzle of how a regulator that is not regulated can govern highly regulated cell cycle transitions. However, the levels and activity of metazoan CAK do not always predict the levels of the activating phosphorylation on the T-loop of Cdks, which is the most relevant output for cell cycle control.

In response to external stimuli, while CAK levels may not change, several groups have reported changes in the levels of T-loop phosphorylation, especially early in the cell cycle. Mitogenic stimulation of fibroblasts (NIH 3T3 cells) turns on mitogen-activated protein kinases (MAPKs) and T-160 phosphorylation of Cdk2, which is blocked by selective MAPK chemical inhibitors [37]. At the time, it was not clear whether the apparent drop in Cdk2 activation upon MAPK inhibition was due to lower CAK activity or inhibition of the nuclear translocation of Cdk2, rendering it inaccessible to the predominantly nuclear Cdk7–cyclinH CAK. However, when Cdk2 was targeted to the nucleus, bypassing the nuclear translocation issue, the activation of MAPK in serum-arrested cells still induced T-160 phosphorylation of Cdk2, which was blocked by adding a selective MAPK inhibitor [38,39]. Hence, an output of mitogenic signaling is Cdk activation through T-loop phosphorylation.

Conversely, there are several reports of anti-mitogenic signaling leading to reduced T-loop phosphorylation, leading to Cdk inactivation, and preventing cells from commencing cell division. For example, transforming growth factor β (TGFβ) signaling was reported to inhibit Cdk2–cyclinE and initiation of DNA replication by inhibiting the phosphorylation of Cdk2 at T-160, while Cdk4/6 activity was maintained [40]. Because the canonical Cdk7–cyclinH CAK activity was unaffected in this context, presumably responsible for the Cdk4/6 activation, the authors proposed a separate CAK activity targeting Cdk2 [40]. Likewise, antimitogenic effects upon inhibiting cholesterol biosynthesis by statin drugs have been attributed to inhibition of the T-160 activating phosphorylation of Cdk2 [41]. Again, since the Cdk7–cyclinH CAK was not inhibited, an additional CAK was postulated [41], along with other models, including selective dephosphorylation of T-160 on Cdk2 [41]. In other cases, it is the activating T-loop phosphorylation of Cdk4 (which functions in G1 before Cdk2) that seems to be inhibited in response to cyclic-AMP (cAMP) signaling that blocks the proliferation of thyroid cancer cells [42]. Remarkably, while Cdk4–cyclinD1 complexes were inhibited by cAMP signaling in these cells, other Cdk–cyclin complexes were not [42], leading the authors to propose that either an unknown CAK targets Cdk4 or some factors bind to Cdk7–cyclinH CAK and govern its specificity toward some Cdks and not others [43]. The above results are intriguing but not always straightforward to interpret, leading to complex models that often invoke mechanisms (e.g., alternate CAKs) that have received limited experimental support over the years. Finally, in addition to the roles of the ‘writers’ (i.e., CAKs) of the activating phosphorylation of Cdks, there are potential roles for the ‘erasers’ of that phosphorylation, such as the KAP [44,45] and other phosphatases, as we will discuss later.

### To divide or not divide – in changing growth conditions

**Figure 1:**
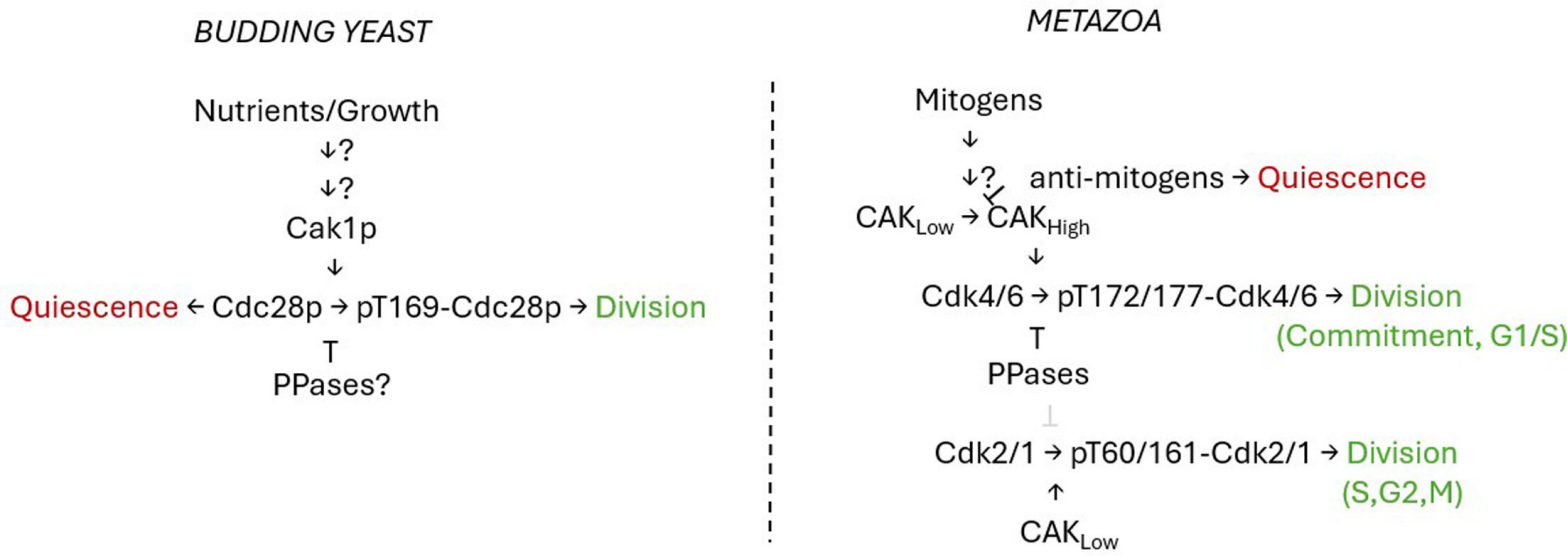
Schematic of a broadly conserved role of CAK in controlling the transition between dividing and quiescent cell states in budding yeast (left) and metazoans (right). Such a unifying view would need to be supported by future work, filling key gaps in knowledge (steps marked by question marks), especially in yeast. See text for details.

As mentioned above, the Cdk7–cyclinH CAK phosphorylates Cdk4/6–cyclinD complexes early in the cell cycle triggering exit from quiescence and transition through G1 [23]. It turns out that the activation of G1 Cdk activity requires maximal CAK levels because phosphorylation is antagonized by competing phosphatases and may be rate-limiting for initiation of cell division [23]. Later, CAK activates the Cdk2 monomer in S phase, followed by Cdk1–cyclin complexes, but those phosphorylations are relatively stable, and CAK activity need not be present at high levels. The above suggests that a higher critical threshold of CAK activity is required for cells to commit to a new round of cell division. As proposed in an elegant model by Schachter et al., a high and sustained CAK activity is required to maintain Cdk activity in G1, driving cells out of quiescence into a mitotic state [23]. Once the cells pass the commitment step, lower CAK activity is needed. This view nicely accounts for the apparent constancy of CAK levels and activity in continuously dividing cells in constant conditions. However, in changing conditions, as cells transition from one state (quiescence) to another (mitotic) in response to external inputs, CAK becomes a critical regulator of the decision to divide (Figure 1).

We argue that this may be a unifying view of CAK’s physiological role in all eukaryotes, including yeast (Figure 1). The clue is the significant drop in Cak1p abundance in the stationary phase, mentioned in one study [25]. Instead of mitogens and growth factors, yeast responds directly to the available nutrients in its environment. Whether the T-loop phosphorylation of the budding yeast Cdk at T169 is also reduced in cells in the stationary phase remains unknown. The mechanism underlying the drop in Cak1p levels in stationary phase cells is also not known. Still, it is probably post-transcriptional since *CAK1* mRNA levels are not regulated as a function of growth in yeast [46]. Furthermore, we note that there is conservation in the type of protein phosphatases (type 2C) that target the T-loop phosphothreonine of Cdks in yeast and humans [47]. Whether any of them (Ptc2p and Ptc3p) has a role in regulating Cdc28p in stationary phase yeast cells is not known. Understanding if and how T-loop phosphorylation and Cak1p levels respond to changes in nutrients in yeast becomes an important task if we are to test the general role of CAK in the decision to divide or not.

Kirova and colleagues recently provided an example of how mammalian cells could use T-loop phosphorylation in response to reactive oxygen species (ROS) production [48]. It turns out that increasing mitochondrial activity and ROS levels leads to the oxidation of a cysteine residue near the T-loop. The oxidized cysteine then blocks the KAP phosphatase from binding Cdk2 and inactivating it by de-phosphorylating it at T160 [48]. The above case illustrates how a metabolic input uses T-loop phosphorylation as an output to govern rates of cell proliferation. In both yeast and mammals, defining the signaling pathways that communicate external nutrient, growth factor, or anti-mitogenic signals to outputs such as CAK or the antagonistic protein phosphatases that together determine the extent of T-loop phosphorylation remains largely unstudied.

**Perspectives**

- T-loop phosphorylation of a conserved threonine residue of cyclin-dependent kinases (Cdk) induces conformational changes that are necessary for productive substrate binding. Cdk-activating kinases (CAKs) are the enzymes responsible for this phosphorylation, but little is known about how the activation of Cdks by phosphorylation is regulated.
- T-loop phosphorylation of Cdks may be a receiver of growth inputs to regulate cell division. This regulation could provide a general mechanism to regulate cell division as cells transition in and out of quiescence or when they are under stress. The down-regulation of CAK levels and T-loop phosphorylation in growth conditions that cannot support cell proliferation may *safeguard* cells from entering a mitotic state.
- Understanding how nutrients and growth factors adjust T-loop phosphorylation and activate the master regulation of cell division will contribute to advancing fundamental knowledge necessary for improving disease treatment. It will enhance our ability to intervene in numerous proliferative diseases.

## Abbreviations

CAKs: Cdk-activating kinases
CTD: C-terminal domain
Cdks: Cyclin-dependent kinases
MAPKs: mitogen-activated protein kinases
TFIIH: transcription factor IIH
